# Temporally and Genetically Discrete Periods of Wheat Sensitivity to High Temperature

**DOI:** 10.3389/fpls.2017.00051

**Published:** 2017-01-25

**Authors:** Henry M. Barber, Martin Lukac, James Simmonds, Mikhail A. Semenov, Mike J. Gooding

**Affiliations:** ^1^School of Agriculture, Policy and Development, University of ReadingReading, UK; ^2^Faculty of Forestry and Wood Sciences, Czech University of Life SciencesPrague, Czechia; ^3^Department of Crop Genetics, John Innes CentreNorwich, UK; ^4^Computational and Systems Biology Department, Rothamsted ResearchHarpenden, UK; ^5^Institute of Biological, Environmental and Rural Sciences, University of AberystwythAberystwyth, UK

**Keywords:** heat stress, meiosis, anthesis, Ppd-D1, Rht, wheat

## Abstract

Successive single day transfers of pot-grown wheat to high temperature (35/30°C day/night) replicated controlled environments, from the second node detectable to the milky-ripe growth stages, provides the strongest available evidence that the fertility of wheat can be highly vulnerable to heat stress during two discrete peak periods of susceptibility: early booting [decimal growth stage (GS) 41–45] and early anthesis (GS 61–65). A double Gaussian fitted simultaneously to grain number and weight data from two contrasting elite lines (Renesansa, listed in Serbia, *Ppd-D1a, Rht8*; Savannah, listed in UK, *Ppd-D1b, Rht-D1b*) identified peak periods of main stem susceptibility centered on 3 (s.e. = 0.82) and 18 (s.e. = 0.55) days (mean daily temperature = 14.3°C) pre-GS 65 for both cultivars. Severity of effect depended on genotype, growth stage and their interaction: grain set relative to that achieved at 20/15°C dropped below 80% for Savannah at booting and Renesansa at anthesis. Savannah was relatively tolerant to heat stress at anthesis. A further experiment including 62 lines of the mapping, doubled-haploid progeny of Renesansa × Savannah found tolerance at anthesis to be associated with *Ppd-D1b, Rht-D1b*, and a QTL from Renesansa on chromosome 2A. None of the relevant markers were associated with tolerance during booting. *Rht8* was never associated with heat stress tolerance, a lack of effect confirmed in a further experiment where *Rht8* was included in a comparison of near isogenic lines in a cv. Paragon background. Some compensatory increases in mean grain weight were observed, but only when stress was applied during booting and only where *Ppd-D1a* was absent.

## Introduction

Improving crop resilience to more frequent extreme weather events is required to maintain or improve crop yields across Europe (Semenov et al., 2014). Wheat, a major contributor to human diet and health (Shewry and Hey, 2015), is particularly susceptible to heat stress around meiosis and anthesis (Barnabas et al., 2008). Yield loss due to heat stress at these growth stages is primarily due to disruption of reproductive processes (Saini and Aspinall, 1982; Saini et al., 1983), as evidenced by a reduction in fertility and grain number (Dolferus et al., 2011). Previous reports on heat stress in wheat usually concern only one of the susceptible timings i.e., meiosis (Saini and Aspinall, 1982; Saini et al., 1984) or anthesis (Tashiro and Wardlaw, 1990; Ferris et al., 1998; Lukac et al., 2012; Pradhan et al., 2012; Steinmeyer et al., 2013; Liu et al., 2016). Fewer studies have attempted to quantify the response to stress at both of these timings: Alghabari et al. (2014) suggest meiosis is the most vulnerable stage, but Prasad and Djanaguiraman (2014) report that it is anthesis that is particularly susceptible. Previous work has often assumed that these growth stages represent two separate, discrete periods of susceptibility but there is currently little evidence to support this. Single experiments on rice and wheat suggest that there may be a period between meiosis and anthesis that is relatively tolerant to heat stress (Satake and Yoshida, 1978; Craufurd et al., 2013), but it is unclear as to the specific growth stages when this tolerance occurs. Genotypic interactions with heat stress timing also require clarification. Although some recent work has compared the heat stress response at anthesis across multiple genotypes (Liu et al., 2016), little work has quantified how genotype influences susceptibility across both stages, even though consecutive exposure of both stages to stress seems likely to occur in field conditions (Wardlaw et al., 1989).

Here, we investigate firstly whether periods of vulnerability to heat stress during reproductive phases can truly be differentiated temporally, in association with growth stage development. Secondly we investigate whether the effect of genotype on heat stress vulnerability interacts with timing of stress. We pay particular attention to the effects of three alleles reported to influence heat stress tolerance and have adaptive significance in wheat grown in European regions with different frequencies and severities of heat stress, namely *Rht8, Ppd-D1a*, and *Rht-D1b* (Worland, 1996; Worland et al., 1998; Rebetzke et al., 2007; Gasperini et al., 2012; Alghabari et al., 2014; Barber et al., 2015; Kowalski et al., 2016; Jones et al., 2017). We also assess associations with the 1BL/1RS translocation (Schlegel and Korzun, 1997) which introduced a number of race-specific disease resistance genes (Snape et al., 2007). The translocation has also been variously associated with increased above ground biomass, spikelet fertility, delayed senescence, and drought tolerance (Villareal et al., 1998; Rajaram, 2001), but there is apparently little information with regards to its influence on heat stress tolerance.

This paper describes the use of 1-day transfers of pot-grown wheat to replicated controlled environments to identify and characterize any periods of heat susceptibility during external growth stages extending from the second node detectable growth stage (GS 32; Zadoks et al., 1974) to the grain milky-ripe stage (GS 77) and hence encompassing meiosis and anthesis (Barber et al., 2015). An initial study compared the Southern European wheat Renesansa (*Ppd-D1a, Rht-D1a, Rht8*) to the UK-adapted wheat Savannah (*Ppd-D1b, Rht-D1b, 1BL/1RS*). Once susceptible growth stages were identified, further experiments compared the heat stress responses of near isogenic lines (NILs) of a Paragon background varying for presence and absence of *Rht8*, and also the responses of a mapping population of 62 doubled haploid progeny of Renesansa × Savannah, at appropriate timings.

## Materials and methods

### Plant material

Savannah has a high yield potential in North West Europe with low bread making quality and was recommended in the UK in 1998. Renesansa, a Serbian winter wheat listed in 1995, has high yield potential and high bread making quality in southern Europe. Sixty-two lines were selected from a recombinant doubled haploid (DH) population of Savannah × Renesansa based on their alleles at Ppd-D1, Rht-D1, 1BL/1RS, and Rht8 (Xgwm261; Simmonds et al., 2006; Snape et al., 2007). NILs varying for the presence and absence of *Rht8*, though both remaining sensitive to photoperiod were developed in a Paragon background (Kowalski et al., 2016). Paragon is a photoperiod sensitive spring wheat that can be also sown in autumn and was first listed in the UK in 1999 with good bread making quality.

### Growing conditions and post-harvest analysis

Plants used in these experiments were grown in pots (180 mm diameter) at the Plant Environment Laboratory at the University of Reading, UK (51 27′ N latitude, 00 56′ W longitude). Each pot contained 2.8 kg of growing media comprising 4:2:4:1 of vermiculite: sand: gravel: compost mixed with Osmocote slow release granules (2 kg m^−3^) containing a ratio of 15:11:13:2 of N:P_2_O_5_:K_2_O:MgO. Seven seeds were sown per pot; thinned to four plants per pot at the two leaf stage. The pots were maintained outside in prevailing conditions (Table 1) under a protective net cage in four randomized blocks with guard pots of wheat placed around the perimeter of experimental blocks. Fungicide was applied as and when required. Pots were watered up to twice daily by an automatic drip irrigation system to maintain field capacity. All treatments consisted of transfers to Saxil growth cabinets, which began between 10:20 and 11:20 h (BST) and remained there for 24 h (16 h day, night time between 22:00 and 06:00 h) before being returned outside to their original randomized block position. Average daily temperature during the treatment period was 14.3°C in 2013/14 and 13.5°C in 2014/15. Two temperature regimes were used in all experiments, day/night temperatures of 20/15 for the control treatment and 35/30°C for the heat stress treatment. Pots were irrigated to field capacity before transfer, but were not irrigated whilst in the cabinets. Eight growth cabinets were used which allowed the two temperature treatments to be replicated for the four blocks. On the day of transfer main stems in each pot were tagged and assessed for growth stage (GS, Zadoks et al., 1974). Pots were weighed immediately before and after transfer to monitor water loss. Main stems and tillers were harvested separately after physiological maturity (GS 89) and dried (48 h at 80°C). Ears and spikelets per ear were counted, after which grain was threshed from ears, then re-dried, weighed, and counted by a Kirby Lester K18 tablet counter.

**Table 1:** **Outside temperatures under which plants were grown in the 2013/14 season**.

**Month (2013/14)**	**Mean of daily minima (°C)**	**Mean of daily maxima (°C)**	**Average mean temperature (°C)**
December	1.9	9.7	5.8
January	2.7	9.4	6.1
February	3.4	9.8	6.6
March	2.9	13.4	8.1
April	5.1	15.1	10.1
May	7.8	17.1	12.5
June	10.5	21.5	16.0
July	12.4	25.0	18.7

### Experiment 1

Experiment 1, sown on the 16th December 2013, comprised a complete factorial of: the two DH parent winter wheat cultivars, Savannah and Renesansa; day of transfer to Saxil growth cabinets (31 separate timings between May 2nd and June 13th 2014); and the two temperature regimes within growth cabinets. Confounding effects associated with temperature included water loss. The mean weight of pots on entry was 3.40 kg, whilst mean weights of pots on withdrawal were 3.19 and 2.98 kg (SED = 0.016) for the 20/15 and 35/30°C treatments, respectively. More detailed studies on the water relations within this growing medium and system suggests that this degree of water loss would equate to 78 and 56% field capacity (FC; oven dry = 0% FC; Gooding et al., 2003), respectively, and that a FC of <70% maintained for 14 days during grain filling was required to reduce grain yield. A further confounded environmental variate was mean relative humidity [73% for 20/15°C and 47% for 35/30°C (SED = 4.4)] whilst in the cabinets.

### Experiment 2

Also sown on the 16th December 2013, the treatment structure comprised a complete factorial design of: three genotypes [Paragon, *Rht8* NIL, and Tall NIL (Kowalski et al., 2016)]; day of transfer to Saxil growth cabinets (5 separate days between 19th May and 10th June 2014) and the two temperature regimes within growth cabinets.

### Experiment 3

Experiment 3 was sown on 3rd December 2014. The treatment structure comprised a complete factorial of 62 DH Lines, three growth stages at transfer to Saxil growth cabinets, and two temperature regimes within growth cabinets. The three timings targeted specific stages of growth: early booting (GS 39–41); mid booting (GS 43–45); and early anthesis (GS 63–65). Due to variable rates of development within a 24 h period, and differential rates of progression, not all lines were transferred within target. Nonetheless, GS at transfer was always recorded.

### Statistical analysis

The primary statistical approach was an appropriate factorial analysis of variance (ANOVA) with a blocking structure of Block/Cabinet/Pot (GenStat 14th edn., VSN International Ltd.). For Experiments 1 and 2, polynomial regressions were fitted across day of transfer to growth cabinet using orthogonal polynomial contrasts in the ANOVA i.e., treatment structure was pol (Day; *n*) ^*^ Temperature ^*^ Genotype, where *n* was the maximum level of polynomial to be fitted. Where quartic effects or deviations from them were significant in Experiment 1, fits were compared with the double Gaussian model (Equation 1) on an *r*^2^ adj basis. The maximal double Gaussian model permits the estimation of two “bell-shaped” curves:

(1) $$\begin{array}{l} Relative\;Effect\;\left(\%\right)=100+b{\left(2\pi s_{1}{}^{2}\right)}^{-0.5}\;e^{-{\left(t-m\right)}^{2}/2s_{1}{}^{2}}\\ \;+\;c{\left(2\pi s_{2}{}^{2}\right)}^{-0.5}\;e^{-{\left(t-n\right)}^{2}/2s_{2}{}^{2})} \end{array}$$

Where: *Relative Effect* is the result at 35°C (day temperature) expressed as a percentage of that achieved at 20°C; *b* and *c* are the size of the two peaks; *m* and *n* are when, in time *t*, they are centered; and *s*_1_ and *s*_2_ are the Gaussian shape factors (standard deviation) for the two peaks. This double Gaussian approach has previously been used to detect other phenologically-dependent responses in wheat time series data sets (Lu et al., 2014). The FITNONLINEAR routine in GENSTAT 14 was used to compare regressions and allow a parsimonious approach to the inclusion of various parameters in the model fits. Additionally, the routine allowed simultaneous fits to different response variates (weighted for the inverses of their variances). Here, it was used to investigate potential compensation in mean grain weights at the time when grain numbers were reduced by heat stress.

Experiment 3 was analyzed by ANOVA with a treatment structure of Genotype × Target Growth Stage × Temperature. A regression analysis was conducted in an attempt to control the effects of varying growth stages within the target GS cohorts. Main and interacting effects of *Rht-D1b, Rht8, Ppd-D1a*, and *1BL/1RS* were tested for their significance in the model (*P* < 0.05). In addition, after correcting for the linear effect of GS within target GS cohort, a QTL analysis was conducted from the effects of the high temperature treatment on individual lines within each target GS. A framework genetic map was constructed from 93 lines of the population as previously described by Snape et al. (2007), containing 107 single sequence repeat (SSR) markers and perfect markers for Ppd-D1, Rht-D1, and 1BL/1RS. Linkage map construction was performed using JoinMap® 3.0 (Kyazma BV) with default settings. Linkage groups were determined using a Divergent log-of-odds (LOD) threshold of 3.0 and genetic distances were computed using the Kosambi regression. The genetic map consisted of 25 linkage groups with 45 unlinked markers. QTL Cartographer 2.5 (North Carolina State University) was used for QTL detection using single marker analysis and composite interval mapping (CIM). Estimates of the additive effects and percentage of total variation for identified QTL were calculated using the multiple interval mapping (MIM) function.

## Results

### Experiment 1

**Figure 1:**
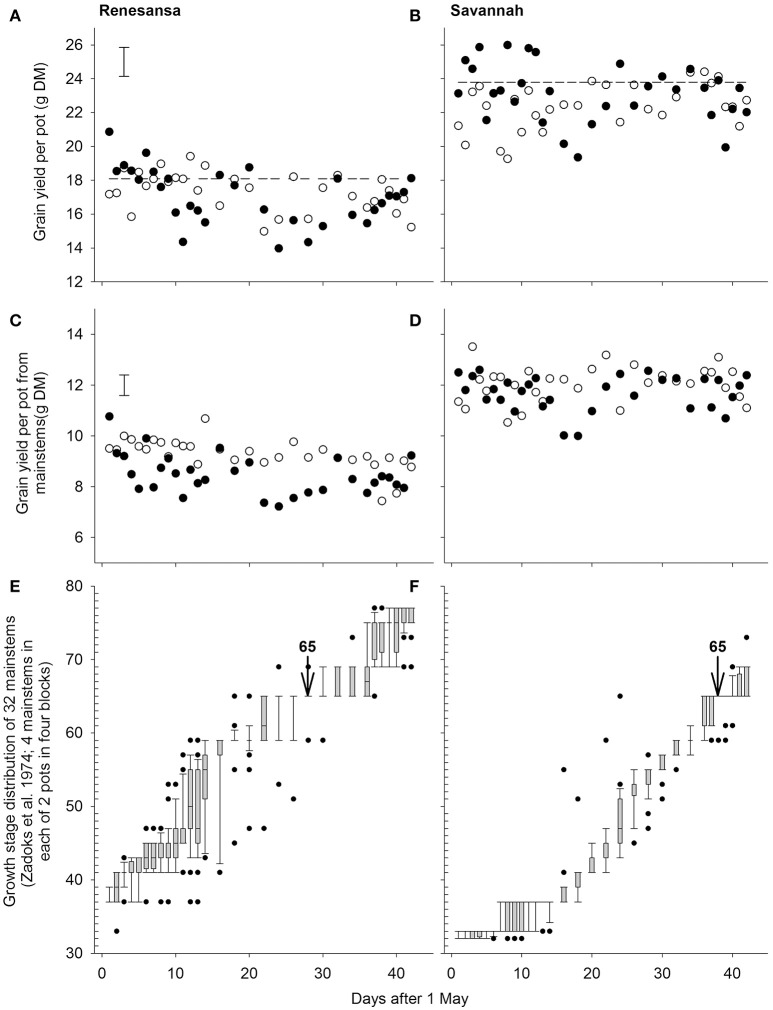
**Effects of wheat cultivar and successive 1-day transfers to controlled environment cabinets at 20/15 (◦) and 35/30°C (•) day/night temperature (16 h day) on grain yield per pot from all stems (A,B)** or only mainstems (**C,D**). Panels **(E,F)** give the growth stage distributions of the mainstems at the time of transfer in to the cabinets (boxes are limited by 25 and 75%, whiskers by 10 and 90%; points are outliers beyond 10 and 90%, and the line within the box is the median where appropriate). S.E.D. (358 d.f.) in **(A,C)** is for comparing temperatures within day and cultivar for both cultivars. Arrows in **(E,F)** denote the assumed timing of growth stage (GS) 65 (Zadoks et al., 1974). Dashed lines in **(A,B)** are the mean yields from eight pots per cultivar left outside.

Grain yield per pot indicated a three factor interaction between day of transfer, temperature and cultivar (*P* = 0.002; deviation from quartic *P* = 0.007; Figures 1A,B). Most of the interaction was due to changes in grain number per pot (*P* < 0.001 for the three factor interaction; deviation from quartic *P* < 0.001), with some modification through partial compensatory increases in mean grain weight, particularly after some of the earlier transfers (e.g., *P* < 0.001 for cubic.Day × Cultivar). There were no (*P* > 0.05) main, or interacting effects, of temperature on ear number per pot (mean for Renesansa and Savannah = 9.2 and 9.5, respectively; S.E.D. = 0.12; 345 d.f.) or spikelet number per ear (Renesansa = 20.3, Savannah = 20.0; S.E.D. = 0.09).

With regards to timing of susceptibility to heat stress, the grain yields from the main stems provided better clarity than the yields from the whole plot, presumably because of the broader spectrum of the growth stages deriving from the tillers (Jones et al., 2017) and as growth stage assessments focussed primarily on main stems. On the main stems, yields of Renesansa appeared to be repeatedly compromised by day transfers to the higher temperature from 6 to 12 May, and again from 22 to 30 May (Figure 1C). In Savannah there was a significant period of susceptibility from the 17 to 21 May, and possibly a second period from 4 to 9 June (Figure 1D). Variation in growth stage amongst mainstems appeared to be greater for Renesansa (Figure 1E) than for Savannah (Figure 1F). Nonetheless, on average, for much of the period of transfers, the growth stage development of Savannah appeared to be about 10 days later than that for Renesansa. This difference could be identified with accuracy at mid anthesis as over 80% of mainstems were scored as at GS 65 on 28 May for Renesansa and on 7 June for Savannah.

**Figure 2:**
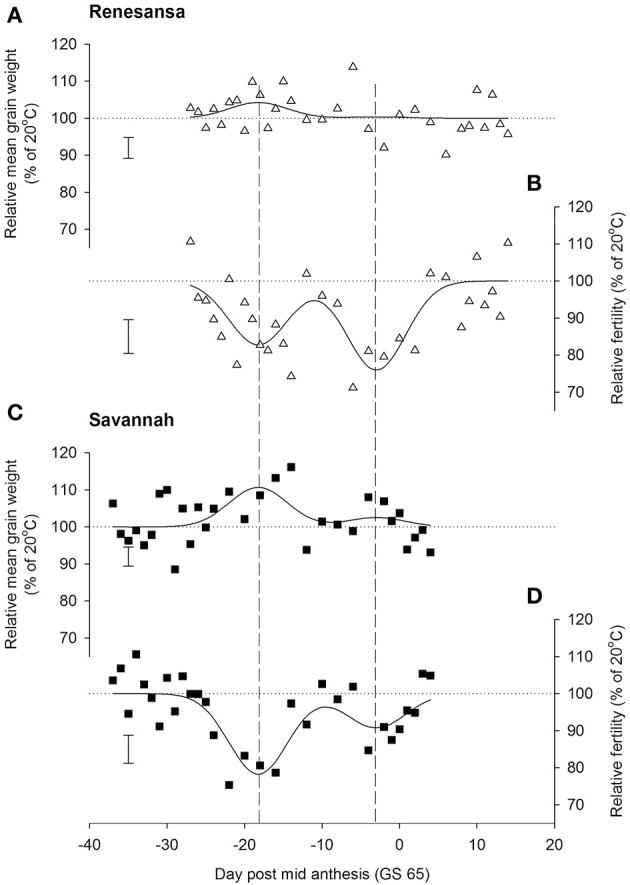
**Effects of increasing day temperature from 20 to 35°C in successive 1-day transfers to controlled environment cabinets on mean grain weight (A,C) and grain numbers (B,D) from main stems of winter wheat, cvs Renesansa (triangles) and Savannah (squares)**. Fits are double Gaussian (Table 1) constrained for peaks to have the same shape (Gaussian S, Equation 1) and timings for the different components and varieties. Error bars are 1 S.E.D. (358 d.f.) for comparison of individual points with the *y* = 100 line.

When Day of transfer was expressed as relative to GS 65, there was strong evidence for two peak timings of susceptibility, but there was no evidence that timing of the peaks for susceptibility varied for the two cultivars, or that the standard deviation of the two peaks varied (Gaussian *s*). With regards to grain numbers on the mainstem (Table 2; Figure 2), a first peak was centered about 18 days before GS 65 when 50% of Renesansa mainstems were at GS 43–45, and 50% of Savannah mainstems were at GS 41–43 (Figure 1). Both cultivars appeared comparatively tolerant of the heat stress during late booting and ear emergence. A second period of susceptibility, however, was detected during late ear emergence and early phases of anthesis, centered on 3 days before GS 65 (Table 2; Figure 2), when most of the ears would have been at GS 61. Grain set in Renesansa appeared equally susceptible to the heat stress during booting and anthesis (Table 2; Figure 2). Grain set in Savannah was significantly more susceptible during booting than at anthesis, but the only time when grain set was significantly compensated by increased mean grain weight was at the earlier timing (Table 2; Figure 2). There was no statistical evidence for compensation for grain set failure through mean grain weight by Renesansa during either period of susceptibility.

**Table 2:** **Parameter values for simultaneous double Gaussian fit (Figure 2) to the effects of increasing day temperature from 20 to 35°C over successive single days for grain yield components on main stems of two cultivars of winter wheat**.

			**Estimate**	**S.e**.
Gaussian shape factor (s, days)			3.71	0.416
Peak position (days relative to GS 65)		Peak 1	–18.2	0.55
		Peak 2	–3.0	0.82
Grain number	Renesansa	Peak 1	–359	66.7
		Peak 2	–491	92.1
	Savannah	Peak 1	–555	92.4
		Peak 2	–231	77.6
Mean grain weight (mg)	Renesansa	Peak 1	17.5	8.5
		Peak 2	2.3	11.8
	Savannah	Peak 1	45.3	12.0
		Peak 2	12.2	10.2

### Experiment 2

**Figure 3:**
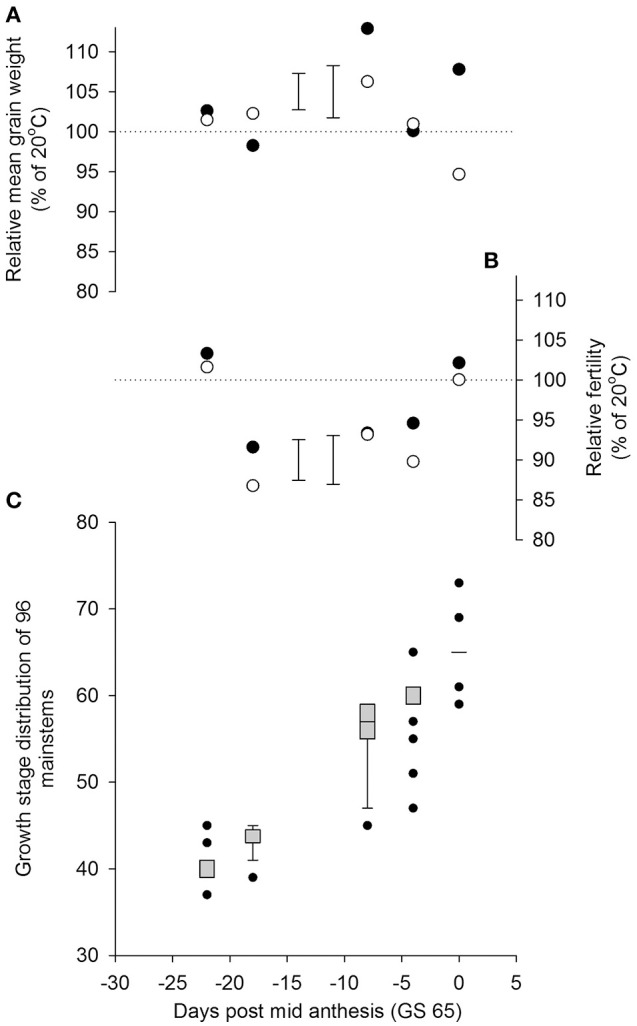
**Effects of increasing day temperature from 20 to 35°C in 1-day transfers to controlled environment cabinets on yield components per pot from main stems of near isogenic lines with (•) and without (◦) ***Rht8*** in a Paragon wheat background**. Error bars in **(A,B)** are S.E.D.s for comparing points without (left) and with (right) *Rht8* with the 100% line. Box-whisker plots (Figure 1 for description) in **(C)** show growth stage distributions of mainstems on day of transfer.

There was a significant interaction between the time of transfer and temperature on mainstem grain number (*P* = 0.005 for Temperature × quadratic Day). As in Experiment 1, a significant reduction in grain numbers from the main stems resulted from a day transfer to 35/30°C rather than 20/15°C, 18 days before mid anthesis (GS 65; Figure 3), whilst the plants were in the early to mid-stages of booting (c. GS 43). There were smaller reductions in grain numbers following heat stress during late ear-emergence and early anthesis, commensurate with the effects on grain numbers of Savannah at similar timings in Experiment 1. Plants appeared tolerant of the higher temperature at the start of booting (c. GS 40) and by mid anthesis (GS 65). There was no statistical evidence in Experiment 2 that reductions in grain numbers were mitigated by increases in mean grain weight; neither was there any evidence that *Rht8* influenced tolerance to heat stress during booting or anthesis (*P* = 0.997 for Temperature × Day × Genotype on mainstem grain numbers).

### Experiment 3

**Figure 4:**
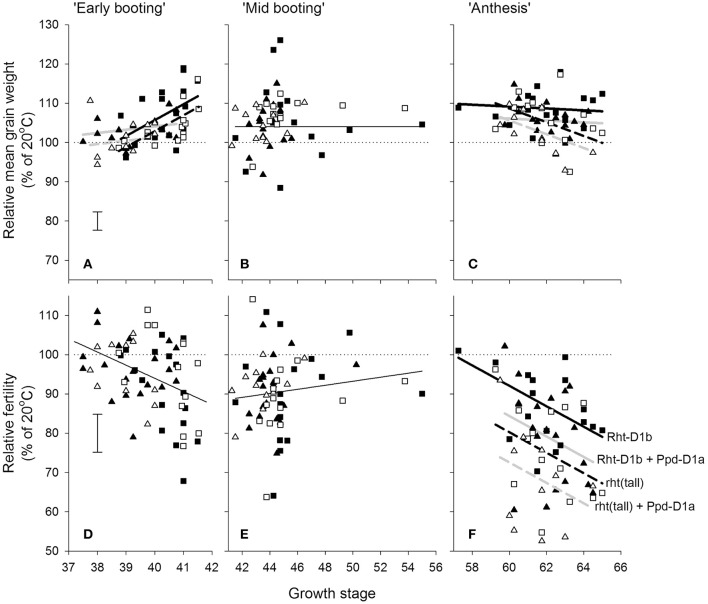
**Effects of increasing day temperature from 20 to 35°C in 1-day transfers to controlled environment cabinets and growth stage** (**A,D** = early booting; **B,E** = mid booting; **C,F** = Anthesis) on mean grain weight **A–C** and grain numbers **(D–F)** from main stems of the doubled haploid progeny of Savannah × Renesansa marked for with (solid symbols) and without (open) *Rht-D1b* and with (triangles) and without (squares) *Ppd-D1a*. Error bars are S.E.D.s for comparing any point with the 100% line. In **A,C,F** lines are fits corresponding to markers as described in **(F)**: with (solid) and without (dashed) *Rht-D1b*; and with (light line) and without (heavy line) *Ppd-D1a*.

Within the doubled haploid population, when using the “target” growth stages for transfer as a fixed effect there was a very highly significant interaction (*P* < 0.001) between temperature, growth stage, and DH line for grain number. When making some allowance for actual growth stages within target stress timings, there was evidence of increasing susceptibility from GS 37 to 41 (Figure 4D) and from GS 59 to 65 (Figure 4F). There was wide variation in susceptibility of lines within the doubled-haploid population, particularly at the mid-booting growth stage (Figures 4B,E). None of this variation was significantly associated with the markers for *Rht8* or the 1BL/1RS translocation. At anthesis, however, main effect associations with both *Rht-D1b* (*P* < 0.001) and *Ppd-D1a* (*P* = 0.006) were significant. *Rht* (tall) and *Ppd-D1a* were associated with increased susceptibility during anthesis (Figure 4F). The QTL analysis confirmed the protective nature of the Savannah alleles (*Rht-D1b* and *Ppd-D1b*), but in addition identified a further, and stronger protective QTL from Renesansa on chromosome 2A (Table 3). None of these alleles could be detected as being protective against heat stress applied during booting. There was however, a weak protective QTL from Renesansa for heat applied during early booting on 2B (nearest marker = Xgwm120; LOD = 1.85; additive effect = −3.75).

**Table 3:** **Quantitative trait loci for relative fertility (%) in response to heat stress during anthesis (grain numbers following 1 day transfer to 35°C as a percentage of that achieved at 20°C)**.

**Chromosome**	**Closest marker**	**LOD**	**Additive effect**	**Source of protecting allele**	**Effect (%)**
2A	*Xgwm448*	7.02	−7.1971	Renesansa	38.1
2D	*Ppd-D1*	2.11	3.7296	Savannah	7.1
4D	*Rht-D1*	3.77	5.2518	Savannah	16.7

In addition to effects on fertility, there was a significant three factor interaction on mean grain weight (*P* = 0.032). Increased mean grain weight at the higher temperature during the early stages of booting (Figure 4A) occurred in the lines not marked for *Ppd-D1a*, and was most evident in lines containing *Rht-D1b*. As anthesis progressed, the higher temperature caused progressively greater reduction in the mean grain weights of lines containing *Ppd-D1a* (Figure 4C).

## Discussion

This study clarifies the effect of heat stress on wheat yield during reproductive development, as well as the influence of growth stage and potentially adaptive genotypic effects. We have identified two discrete periods at which grain set in wheat is susceptible to high temperature: the first in early to mid-booting presumably commensurate with susceptible meiotic stages (Barber et al., 2015) and the second during the early phases of anthesis. We have demonstrated that genotypic effects on tolerance to heat stress vary with the particular period of vulnerability.

Reductions in grain number due to heat stress caused by reduced fertility found across all experiments in this study are in agreement with previous work (Saini and Aspinall, 1982; Ferris et al., 1998; Dolferus et al., 2011; Liu et al., 2016). There is some evidence to suggest that grain size can increase and partially compensate for losses caused by abiotic stresses (Semenov et al., 2014), however this is mostly confined to the booting period of susceptibility and was not consistently observed across genotypes. Grain size increases found at booting but not at anthesis support the lack of grain size compensation found by Liu et al. (2016). This variation in compensatory increases in mean grain weight over genotype and growth stage should be accounted for when attempting to improve the response of crop models to abiotic stress (Stratonovitch and Semenov, 2015; Liu et al., 2016). Consistent with previous literature, the peak periods of susceptibility appear to be early to mid-booting (Saini and Aspinall, 1982; Alghabari et al., 2014) and early flowering (Ferris et al., 1998; Craufurd et al., 2013; Prasad and Djanaguiraman, 2014). There is some evidence to suggest that the period between meiosis and anthesis appears to be relatively tolerant to short durations of heat stress: similar to what has been observed in rice (Satake and Yoshida, 1978, 1981; Craufurd et al., 2013), with indications that this could also be true in wheat (Prasad and Djanaguiraman, 2014). Responses to heat stress are strongly influenced by genotype, as shown by variation within these experiments, especially between Savannah and Renesansa. Genotypic differences, especially at anthesis, as observed here, have been identified previously (Stone and Nicolas, 1994; Alghabari et al., 2014; Lobell et al., 2015; Liu et al., 2016). This suggests that there is potential for identifying heat tolerant traits within the current genetic diversity of wheat, which will be crucial for crop production in future climates (Godfray et al., 2010; Semenov et al., 2014).

It is necessary to acknowledge the possible confounding effects between heat stress tolerance and water deficit (Barnabas et al., 2008; Alghabari et al., 2014) in these experiments. However, the deficits below FC reported here at the end of pot transfer, and the durations over which significant deficits could have occurred, are considered to be relatively minor compared with the results from experiments with longer periods of stress (Gooding et al., 2003; Alghabari et al., 2014). Nonetheless, booting is known to be a period particularly susceptible to drought (Barber et al., 2015) and future work on identifying tolerant traits to abiotic stresses will require consideration of the combination of drought and heat stress.

There has previously been some suggestion that the semi dwarfing allele *Rht8*, commonly found in southern European genotypes of wheat (Worland, 1996; Gasperini et al., 2012), could also increase tolerance to heat and drought stress compared to other semi dwarfing alleles (Alghabari et al., 2014). However, our study found no effect of *Rht8* on susceptibility to heat stress. This suggests that even in future climates, *Rht8* would not be of benefit to northern European genotypes due to its lower yield in comparison to other semi dwarfing alleles (Rebetzke et al., 2007). Furthermore, *Ppd-D1a*, to which *Rht8* is closely linked (Gasperini et al., 2012) was shown to increase susceptibility to heat stress. Photoperiod insensitivity caused by the allele *Ppd-D1a*, a mechanism used to avoid abiotic stress (Gomez et al., 2014), is widely considered to be a beneficial trait in future climates due to reducing thermal time to senescence (Barber et al., 2015), thereby avoiding late season heat and drought stress. It was also suggested by Jones et al. (2017) that the increase in flowering duration associated with *Ppd-D1a* would add further resilience by increasing diversity of flowering timing within a field. However, the increase in susceptibility to heat stress associated with this allele, as well as lower overall grain yield in non-stressed seasons (Addisu et al., 2010) casts doubt over the benefits that *Ppd-D1a* might bring under future northern European climates. Although the introduction of *Rht-D1b* in to Northern European wheats has increased yield through increased harvest index and reduced lodging in fertile conditions (Flintham et al., 1997), it has also been associated with some negative traits, including decreases in fertility (Law et al., 1981). Preliminary work by Law and Worland (1985) suggested that the decrease in GA sensitivity caused by *Rht-D1b* increases susceptibility to heat stress. This is supported by later work in other cereals, such as barley, which shows that reducing sensitivity to GA increases susceptibility to heat stress (Vettakkorumakankav et al., 1999; summary provided by Maestri et al., 2002). However, our study shows evidence to the contrary. Here, *Rht-D1b* was associated with greater tolerance of high temperatures at anthesis than the other alleles associated with stature. In particular, the tall allele at the *Rht-D1* locus was associated with susceptibility to heat stress at anthesis. This contrasts with the effects of *Rht-D1* dwarfing alleles in some, but not all, backgrounds reported by Alghabari et al. (2014). We have found no genetic explanation for the poor performance of the Northern European genotype at booting. However, this can likely be attributed to the lack of selection pressure previously on breeding programmes for this trait.

With respect to the QTL analyses, others have also found regions on chromosomes on 2A and 2B to be associated with differential responses to heat stress (Mason et al., 2010; Talukder et al., 2014). Given the strength of the protective effect associated with the QTL on 2A further investigation is warranted for alleles in the relevant region from Renesansa. What is very clear from this study is that alleles and QTL detected as being associated with heat stress tolerance is highly dependent on the precise growth stage of the plant when excessive heat is experienced.

## Conclusions

In conclusion, this paper provides the strongest existing evidence that the key phases susceptible to heat stress at booting and anthesis in wheat are discrete and that genotypes vary with regards to the most susceptible growth stage. Periods of susceptibility are repeatedly observed during GS 41–45 and again from GS 61–65. In the prevailing conditions (mean daily temperature 14.3°C) periods of peak susceptibility could be separated by 15 days. We found no evidence that the southern European semi dwarfing allele *Rht8* adds tolerance to heat stress within NILs or a DH population. In contrast, the north European allele *Rht-D1b* was associated with increased tolerance to heat stress at anthesis. The photoperiod insensitivity allele *Ppd-D1a* was also found to be linked to increased susceptibility to heat stress.

## Author contributions

HB, MG, and MS contributed to experimental design, HB and MG conducted analysis on the data with assistance from ML on interpretation of the data, whilst JS conducted QTL and genetic analysis. HB and MG drafted the work with revisions from MS, ML, and JS. HB, MG, MS, ML, and JS approve of the final version of the manuscript and all agree to be accountable for all aspects of the work.

### Conflict of interest statement

The authors declare that the research was conducted in the absence of any commercial or financial relationships that could be construed as a potential conflict of interest.

## References

[B1] Addisu M. Snape J. W. Simmonds J. R. Gooding M. J. (2010). Effects of reduced height (Rht) and photoperiod insensitivity (Ppd) alleles on yield of wheat in contrasting production systems. Euphytica 172, 169–181. 10.1007/s10681-009-0025-2

[B2] Alghabari F. Lukac M. Jones H. E. Gooding M. J. (2014). Effect of Rht alleles on the tolerance of wheat grain set to high temperature and drought stress during booting and anthesis. J. Agron. Crop Sci. 200, 36–45. 10.1111/jac.12038

[B3] Barber H. M. Carney J. Alghabari F. Gooding M. J. (2015). Decimal growth stages for precision wheat production in changing environments? Ann. Appl. Biol. 166, 355–371. 10.1111/aab.12207

[B4] Barnabas B. Jaeger K. Feher A. (2008). The effect of drought and heat stress on reproductive processes in cereals. Plant Cell Environ. 31, 11–38. 10.1111/j.1365-3040.2007.01727.x 17971069

[B5] Craufurd P. Q. Vadez V. Jagadish S. V. K. Prasad P. V. V. Zaman-Allah M. (2013). Crop science experiments designed to inform crop modeling. Agric. For. Meteorol. 170, 8–18. 10.1016/j.agrformet.2011.09.003

[B6] Dolferus R. Ji X. Richards R. A. (2011). Abiotic stress and control of grain number in cereals. Plant Sci. 181, 331–341. 10.1016/j.plantsci.2011.05.015 21889038

[B7] Ferris R. Ellis R. H. Wheeler T. R. Hadley P. (1998). Effect of high temperature stress at anthesis on grain yield and biomass of field-grown crops of wheat. Ann. Bot. 82, 631–639. 10.1006/anbo.1998.0740

[B8] Flintham J. E. Borner A. Worland A. J. Gale M. D. (1997). Optimizing wheat grain yield: effects of Rht (giberellin-insensitive) dwarfing genes. J. Agri. Sci. Camb. 128, 11–25. 10.1017/S0021859696003942

[B9] Gasperini D. Greenland A. Hedden P. Dreos R. Harwood W. Griffiths S. (2012). Genetic and physiological analysis of Rht8 in bread wheat: an alternative source of semi-dwarfism with a reduced sensitivity to brassinosteroids. J. Exp. Bot. 63, 4419–4436. 10.1093/jxb/ers138 22791821 PMC3421992

[B10] Godfray H. C. J. Beddington J. R. Crute I. R. Haddad L. Lawrence D. Muir J. F.. (2010). Food security: the challenge of feeding 9 billion people. Science 327, 812–818. 10.1126/science.1185383 20110467

[B11] Gomez D. Vanzetti L. Helguera M. Lombardo L. Fraschina J. Miralles D. J. (2014). Effect of Vrn-1, Ppd-1 genes and earliness *per se* on heading time in Argentinean bread wheat cultivars. Field Crop. Res. 158, 73–81. 10.1016/j.fcr.2013.12.023

[B12] Gooding M. J. Ellis R. H. Shewry P. R. Schofield J. D. (2003). Effects of restricted water availability and increased temperature on the grain filling, drying and quality of winter wheat. J. Cereal Sci. 37, 295–309. 10.1006/jcrs.2002.0501

[B13] Jones H. E. Lukac M. Brak B. Martinez-Eixarch M. Alhomedhi A. Gooding M. J. (2017). Photoperiod sensitivity affects flowering duration in wheat. J. Agri. Sci. 155, 32–43. 10.1017/S0021859616000125

[B14] Kowalski A. M. Gooding M. Ferrante A. Slafer G. A. Orford S. Gasperini D.. (2016). Agronomic assessment of the wheat semi-dwarfing gene *Rht8* in contrasting nitrogen treatments and water regimes. Field Crops. Res. 191, 150–160. 10.1016/j.fcr.2016.02.026 27212788 PMC4862442

[B15] Law C. N. Snape J. W. Worland A. J. (1981). Reduced Fertility of Wheat Associated with Rht3. Plant Breeding Institute Annual Report 1980, Plant Breeding Institute, Cambridge.

[B16] Law C. N. Worland A. J. (1985). An Effect of Temperature on the Fertility of Wheats Containing the Dwarfing Genes Rht1, Rht2 and Rht3. Cambridge: Plant Breeding Institute.

[B17] Liu B. Asseng S. Liu L. Tang L. Cao W. Zhu Y. (2016). Testing the responses of four wheat crop models to heat stress at anthesis and grain filling. Glob. Change Biol. 22, 1890–1903. 10.1111/gcb.13212 26725507

[B18] Lobell D. B. Hammer G. L. Chenu K. Zheng B. Mclean G. Chapman S. C. (2015). The shifting influence of drought and heat stress for crops in northeast Australia. Glob. Change Biol. 21, 4115–4127. 10.1111/gcb.13022 26152643

[B19] Lu L. L. Wang C. Z. Guo H. D. Li Q. T. (2014). Detecting winter wheat phenology with SPOT-VEGETATION data in the North China Plain. Geocarto Int. 29, 244–255. 10.1080/10106049.2012.760004

[B20] Lukac M. Gooding M. J. Griffiths S. Jones H. E. (2012). Asynchronous flowering and within-plant flowering diversity in wheat and the implications for crop resilience to heat. Ann. Bot. 109, 843–850. 10.1093/aob/mcr308 22186277 PMC3286278

[B21] Maestri E. Klueva N. Perrotta C. Gulli M. Nguyen H. T. Marmiroli N. (2002). Molecular genetics of heat tolerance and heat shock proteins in cereals. Plant Mol. Biol. 48, 667–681. 10.1023/A:1014826730024 11999842

[B22] Mason R. E. Mondal S. Beecher F. W. Pacheco A. Jampala B. Ibrahim A. M. H. (2010). QTL associated with heat susceptibility index in wheat (*Triticum aestivum* L.) under short-term reproductive stage heat stress. Euphytica 174, 423–436. 10.1007/s10681-010-0151-x

[B23] Pradhan G. P. Prasad P. V. V. Fritz A. K. Kirkham M. B. Gill B. S. (2012). High temperature tolerance in aegilops species and its potential transfer to wheat. Crop Sci. 52, 292–304. 10.2135/cropsci2011.04.0186

[B24] Prasad P. V. V. Djanaguiraman M. (2014). Response of floret fertility and individual grain weight of wheat to high temperature stress: sensitive stages and thresholds for temperature and duration. Funct. Plant Biol. 41, 1261–1269. 10.1071/FP14061 32481075

[B25] Rajaram S. (2001). Prospects and promise of wheat breeding in the 21st century. Euphytica 119, 3–15. 10.1023/A:1017538304429

[B26] Rebetzke G. J. Richards R. A. Fettell N. A. Long M. Condon A. G. Forrester R. I. (2007). Genotypic increases in coleoptile length improves stand establishment, vigour and grain yield of deep-sown wheat. Field Crop. Res. 100, 10–23. 10.1016/j.fcr.2006.05.001

[B27] Saini H. S. Aspinall D. (1982). Abnormal sporogenesis in wheat (*Triticum-Aestivum* L) induced by short periods of high-temperature. Ann. Bot. 49, 835–846.

[B28] Saini H. S. Sedgley M. Aspinall D. (1983). Effect of heat-stress during floral development on pollen-tube growth and ovary anatomy in wheat (*Triticum-Aestivum*-L). Aust. J. Plant Physiol. 10, 137–144. 10.1071/PP9830137

[B29] Saini H. S. Sedgley M. Aspinall D. (1984). Developmental anatomy in wheat of male-sterility induced by heat-stress, water deficit or abscisic-acid. Aust. J. Plant Physiol. 11, 243–253. 10.1071/PP9840243

[B30] Satake T. Yoshida H. (1978). High temperature-induced sterility in indica rices at flowering. Jpn. J. Crop Sci. 47, 6–17. 10.1626/jcs.47.6

[B31] Satake T. Yoshida H. (1981). High temperature stress in rice. IRRI Res. Pap. Ser. 67, 1–15.

[B32] Schlegel R. Korzun V. (1997). About the origin of 1RS.1BL wheat-rye chromosome translocations from Germany. Plant Breed. 116, 537–540. 10.1111/j.1439-0523.1997.tb02186.x

[B33] Semenov M. A. Stratonovitch P. Alghabari F. Gooding M. J. (2014). Adapting wheat in Europe for climate change. J. Cereal Sci. 59, 245–256. 10.1016/j.jcs.2014.01.006 24882934 PMC4026126

[B34] Shewry P. R. Hey S. J. (2015). The contribution of wheat to human diet and health. Food Energy Secur. 4, 178–202. 10.1002/fes3.64 27610232 PMC4998136

[B35] Simmonds J. R. Leverington-Waite M. Wang Y. Greenland A. Snape J. W. (2006). Discovering QTL Controlling Yield and Yield Components in Wheat., John Innes Centre. Norwich: European Cereals Genetics Cooperative.

[B36] Snape J. W. Foulkes M. J. Simmonds J. Leverington M. Fish L. J. Wang Y. (2007). Dissecting gene × environmental effects on wheat yields via QTL and physiological analysis. Euphytica 154, 401–408. 10.1007/s10681-006-9208-2

[B37] Steinmeyer F. T. Lukac M. Reynolds M. P. Jones H. E. (2013). Quantifying the relationship between temperature regulation in the ear and floret development stage in wheat (*Triticum aestivum* L.) under heat and drought stress. Funct. Plant Biol. 40, 700–707. 10.1071/FP12362 32481142

[B38] Stone P. J. Nicolas M. E. (1994). Wheat cultivars vary widely in their responses of grain yield and quality to short periods of post-anthesis heat stess. Aust. J. Plant Physiol. 21, 887–900. 10.1071/PP9940887

[B39] Stratonovitch P. Semenov M. A. (2015). Heat tolerance around flowering in wheat identified as a key trait for increased yield potential in Europe under climate change. J. Exp. Bot. 66, 3599–3609. 10.1093/jxb/erv070 25750425 PMC4463804

[B40] Talukder S. K. Babar M. A. Vijayalakshmi K. Poland J. Prasad P. V. V. Bowden R.. (2014). Mapping QTL for the traits associated with heat tolerance in wheat (*Triticum aestivum*.L.). BMC Genet. 15:97 10.1186/s12863-014-0097-4 25384418 PMC4234900

[B41] Tashiro T. Wardlaw I. F. (1990). The response to high-temperature shock and humidity changes prior to and during the early stages of grain development in wheat. Aust. J. Plant Physiol. 17, 551–561. 10.1071/PP9900551

[B42] Vettakkorumakankav N. N. Falk D. Saxena P. Fletcher R. A. (1999). A crucial role for gibberellins in stress protection of plants. Plant Cell Physiol. 40, 542–548. 10.1093/oxfordjournals.pcp.a029575

[B43] Villareal R. L. Banuelos O. Mujeeb-Kazi A. Rajaram S. (1998). Agronomic performance of chromosomes 1B and T1BL.1RS near-isolines in the spring bread wheat Seri M82. Euphytica 103, 195–202. 10.1023/A:1018392002909

[B44] Wardlaw I. F. Dawson I. A. Munibi P. Fewster R. (1989). The tolerance of wheat to high temperatures during reproductive growth. 1 survey procedures and general response patterns. Aust. J. Agric. Res. 1, 1–13. 10.1071/AR9890001

[B45] Worland A. J. (1996). The influence of flowering time genes on environmental adaptability in European wheats. Euphytica 89, 49–57. 10.1007/BF00015718

[B46] Worland A. J. Borner A. Korzun V. Li W. M. Petrovic S. Sayers E. J. (1998). The influence of photoperiod genes on the adaptability of European winter wheats (Reprinted from Wheat: prospects for global improvement, 1998). Euphytica 100, 385–394. 10.1023/A:1018327700985

[B47] Zadoks J. C. Chang T. T. Konzak C. F. (1974). Decimal code for growth stages of cereals. Weed Res. 14, 415–421. 10.1111/j.1365-3180.1974.tb01084.x

